# Rationale and design of a multicenter randomized controlled trial on a 'minimal intervention' in Dutch army personnel with nonspecific low back pain [ISRCTN19334317]

**DOI:** 10.1186/1471-2474-5-40

**Published:** 2004-11-09

**Authors:** Pieter H Helmhout, Chris C Harts, J Bart Staal, Rob A de Bie

**Affiliations:** 1 Department of Training Medicine and Training Physiology, Occupational Health & Safety Service Royal Netherlands Army, P.O. Box 90004, 3509 AA Utrecht, The Netherlands; 2 Department of Epidemiology, Maastricht University, P.O. Box 616, 6200 MD Maastricht, The Netherlands

## Abstract

**Background:**

Researchers from the Royal Netherlands Army are studying the potential of isolated lumbar extensor training in low back pain in their working population. Currently, a randomized controlled trial is carried out in five military health centers in The Netherlands and Germany, in which a 10-week program of not more than 2 training sessions (10–15 minutes) per week is studied in soldiers with nonspecific low back pain for more than 4 weeks. The purpose of the study is to investigate the efficacy of this 'minimal intervention program', compared to usual care. Moreover, attempts are made to identify subgroups of different responders to the intervention.

**Methods:**

Besides a baseline measurement, follow-up data are gathered at two short-term intervals (5 and 10 weeks after randomization) and two long-term intervals (6 months and one year after the end of the intervention), respectively. At every test moment, participants fill out a compound questionnaire on a stand-alone PC, and they undergo an isometric back strength measurement on a lower back machine.

Primary outcome measures in this study are: self-assessed degree of complaints and degree of handicap in daily activities due to back pain. In addition, our secondary measurements focus on: fear of movement/(re-) injury, mental and social health perception, individual back extension strength, and satisfaction of the patient with the treatment perceived. Finally, we assess a number of potential prognostic factors: demographic and job characteristics, overall health, the degree of physical activity, and the attitudes and beliefs of the physiotherapist towards chronic low back pain.

**Discussion:**

Although a substantial number of trials have been conducted that included lumbar extension training in low back pain patients, hardly any study has emphasized a minimal intervention approach comparable to ours. For reasons of time efficiency and patient preferences, this minimal sports medicine approach of low back pain management is interesting for the population under study, and possibly for comparable working populations with physical demanding job activities.

## Background

### Treatment of low back pain in the military setting: a 'minimal intervention approach'

For the last ten years, clinical researchers from the Royal Netherlands Army (RNLA) have studied the potential of physical training modalities in preventing and alleviating nonspecific low back pain (LBP) in their working population.

Many military and civilian job functions in the RNLA involve heavy manual material handling and, therefore, spine-loading activities. In general, the incidence of back problems is higher in physically demanding tasks than in sedentary activities [1]. Concordantly, the incidence of LBP in the RNLA is high. Acute LBP is the primary reason for soldiers to visit the general practitioner at a military health center. Chronic nonspecific LBP, defined as having complaints for at least 12 weeks, is one of the three most diagnosed disorders during consulting hours of Dutch military company doctors, and takes on average 15% of their weekly consulting hours time.

Currently, there is strong evidence that exercise therapy is more effective than usual care [2]. Exercise therapy is a major part of the standard treatment by physiotherapists in the RNLA, involving an active role of the patient. In the experience of our health professionals (practitioners and physiotherapists), this active approach fits in well with the attitudes and beliefs of the target population: soldiers are taught to be aware of their physical abilities and limitations from the moment they enlist for the army. After all, recruits who do not successfully complete their basic training cannot progress on to a career as a soldier.

The specific character of military tasks nowadays, e.g. (preparation for) crisis management operations abroad, interferes with the schedules of rehabilitation programs for back-injured soldiers which, in general, take several days per week over a considerate number of consecutive weeks. Therefore, the search for effective and (time-) efficient exercise therapy protocols has led us to a specific form of lumbar extension training. Each training session consists of no more than 5 to 10 minutes training of the isolated lumbar extensors on a special training device.

### Arguments for this study

For a number of years, we have experience with high-intensive, isolated training of the lumbar extensors in military personnel with nonspecific LBP, by using a special training device. In this a sports medicine approach is followed, partly according to established exercise protocols [3,4], in which three key principles are emphasized: (1) isolation of the lumbar extensors through fixation of the pelvis and thighs; (2) training in the individual's full range of motion; (3) avoiding 'sticking points' in the training load – i.e. points in the range of motion in which a relatively high resistance is experienced – by tuning the load curve of the weight stack to the individual's strength curve.

In individual cases, we observed satisfying to sometimes excellent results in terms of pain relief and functional restoration, when giving a training stimulus of no more than 5 to 10 minutes (1 to 2 training sessions) per week. These findings, however, need to be confirmed in a randomized controlled trial.

Four main reasons led to the choice of doing research on the efficacy of this sports medicine approach for our working population with LBP.

First, recent systematic reviews indicate that exercise therapy is a successful approach for the restoration of chronic and recurrent LBP, at least in the short term [2,3]. However, higher quality studies generally show a lack of treatment specificity of different exercise modalities, e.g. aerobic exercises, strength and endurance reconditioning or mobilizing exercises [4,5].

Moreover, controversy remains regarding the impact of a training stimulus, in terms of intensity, duration and frequency, on the reduction of LBP. Different explanations for this lack of specificity are given in the literature, such as non-specific, more centrally induced training effects, e.g. a shift in pain perception [5], or large heterogeneity in the chosen study populations [6]. If, indeed, no specific dimension or type of exercise therapy is superior to one another in producing optimal therapeutic outcomes, other aspects are more relevant when introducing an intervention program, such as: treatment affinity, expectation and compliance of patient and provider, costs, facilities, and personnel capacity.

From this perspective, back strength and endurance training in CLBP patients with the use of training devices is an interesting concept for our military population. RNLA personnel are, from their very first initial military education, used to participate in physical exercise programs, including progressive resistance training on exercise machines. The RNLA is well equipped with an extensive line of modern fitness devices on all major military locations throughout the country, including state-of-the-art lower back machines. Moreover, protocolized treatment sessions with our training device take no longer than 5 to 10 minutes once or twice a week from both patient and provider, compared to (on an average) 30 minutes in regular treatment sessions. We expect this time efficiency to be highly appreciated by our personnel, who work in a typical military culture of "running into extremes": relatively quiet (maintenance) periods on the military base are interspersed with extremely busy periods shortly before and during out-of-area operations. For several target groups, longstanding and time-consuming rehabilitation programs are out of the question. For instance, recruits who drop out of their initial training because of (back) injuries, need to return as quick as possible to prevent a stagnation in their military career. For soldiers standing by for military operations, everything revolves around the mission when being commanded to be prepared within the next weeks.

A second reason for our approach is that the majority of studies on LBP management consist of multimodal interventions, which include physical, behavioral, educational and/or ergonomic elements. To obtain a better view on the (relative) efficacy of either of these concepts, unimodal intervention programs like ours need to be evaluated [7,8]. Besides, we strongly believe that exercise as the primary entrance for restoring back function has a wide span of treatment effects, including improvements for cognitive and/pr behavioral variables. Although exercise has a primary goal of improving functioning of targeted tissues, successful completion of exercise protocols in the presence of chronic pain may for example lead to a reduction in pain-related fears. As standardized exercise on a training machine is based on measured performance (number of kilograms and repetitions), patients are continuously given numerical feedback regarding their increasing physical capacities [9]. An increased awareness of improving physical capacities may draw their attention away from pain and suffering.

Third, the choice for a particular intervention approach depends in many cases on the stage and severity of the back problem, the extent to which psychosocial aspects are involved, and the needs and preferences of the patient. For instance, behavioral therapy is mainly focused on issues that are prevalent in chronic patients, such as low feelings of self-control or fear of movement/(re-)injury. Since the population of the present study, military employees of the RNLA, is a working population with mostly short-term, intermittent and moderately severe LBP, we chose to apply a more physical approach. As we have seen in our previous research (see the next paragraph), this links well with the health perceptions of our target population, in which perceived health problems were not severe and much more focused on physical than on mental aspects.

Fourth, the efficacy of isolated extension training in chronic back patients has been studied by several other research groups as well [10]. Although promising results were reported regarding lumbar strength improvements and pain relief, several methodological shortcomings hinder solid interpretation of these findings. Most problems encountered were a small sample size, lack of randomization, lack of long-term follow-up results, variation in study populations (e.g. healthy volunteers, employees receiving worker's compensation), and inadequate or missing control groups [7,10-14]. In a review on lumbar extension training with MedX-equipment in LBP patients, Miltner et al [8] conclude that more controlled studies are needed "to delineate further the role of isolated lumbar extension exercise for the treatment of LBP and to test the efficacy compared to other methods of care."

### Earlier research on our minimal intervention approach

Especially in recent years, we have scientifically studied the potential of our sports medicine approach. In two previous trials we compared the efficacy of a high-intensive, progressive resistance training program of the isolated lumbar extensors, with a low-intensive, non-progressive program of the same extend, in a group of workers with nonspecific LBP. Total intervention time of both 'minimal intervention programs' was limited to 14 sessions of 5 to 10 minutes, over a period of 12 weeks (1^st ^trial) or 8 weeks (2^nd ^trial).

In the first trial, we were unable to demonstrate that either of the two training programs was superior in alleviating back complaints [15]. However, the magnitude of the improvements in back function found in this study were in line with those reported in other studies, which used more extended (multimodal) exercise programs. Therefore, it would be interesting to compare the efficacy of our minimal intervention program with the usual care RNLA personnel with nonspecific LBP.

Moreover, the results of our first trial indicated that some individuals with LBP might benefit more from an aggressive approach, showing a trend towards a higher improvement rate (self-assessed percentage decrease in complaints) directly after the 'minimal intervention' treatment, as well as a higher compliance to the treatment and a higher willingness to participate in physical exercise on the longer term. In the present multicenter study, we aim at identifying relevant subgroups of patients that show higher success rate due to this training approach.

## Methods

### Study design and population

In a randomized, single-blinded multicenter trial, we evaluate the efficacy of progressive, isolated resistance training of the lumbar extensor muscles, compared to the usual care. The trial started in April 2002; data will be collected till the end of 2005. The source population (n = 23,000) consists of military employees of the RNLA. Our in- and exclusion criteria are listed in table 1.

**Figure 1:**
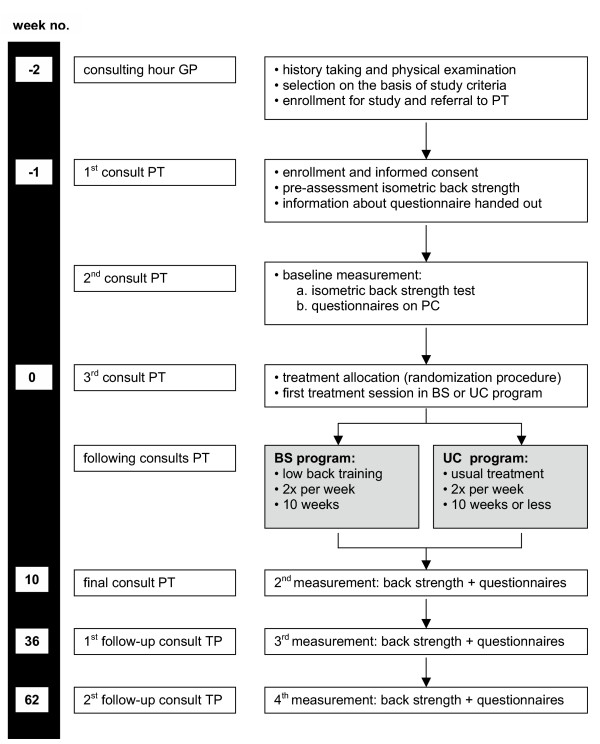
Flow chart of the different phases of the multicenter trial

Recruitment of participants takes place during regular office hours of the military general practitioner. A brief outline of the study design is presented in Figure 1.

### Study sites

Almost every military location in the Netherlands has a health center, in which general practitioners, dentists and physiotherapists give primary care to military personnel of the RNLA. For the present study we selected five military health centers on the basis of: (1) representing a major part of the total military population; (2) holding at least two full-time physiotherapists, and (3) the willingness to unconditionally participate in the project.

The selected military health centers include the following locations:

• Amersfoort, in the middle-east of the Netherlands: approximately 15% younger soldiers on stand-by for military operations abroad (18–25 years), 25% military instructors (35+ years), 10% staff personnel (40+ years), and 46% civilian workers from supporting units (40+ years);

• Den Haag, in the west of the Netherlands: approximately 90% older staff personnel (40+ years) at office work;

• Oirschot, in the south of the Netherlands: approximately 75% younger soldiers in their initial military education or on stand-by for military operations abroad (18–25 years), 25% military instructors and staff personnel (35+ years);

• Schaarsbergen, in the south-east of the Netherlands: approximately 65% younger soldiers in their initial military education or on stand-by for military operations abroad (18–25 years), and 30% staff personnel (35+ years);

• Seedorf, in the north-west of Germany (part of the 1 German/Netherlands Corps): approximately 65% younger soldiers on stand-by for military operations abroad (18–25 years), 20% staff personnel (35+ years), and 10% civilian workers from supporting units (40+ years).

### Study population

#### Recruitment, enrollment, and randomization

All general practitioners at each of the selected study centers identify potential subjects from among their clinic's patients, according to the aforementioned criteria. Each subject is submitted to regular history taking and physical examination by the physician; checklists with standard elements for LBP have been provided to all physicians. If eligible, patients are informed about the study. All relevant information from the intake is written down in a referral; a visit to one of the physiotherapists of the center is planned within the next days.

At the first visit to the physiotherapist, patients receive further information about the trial. A pre-assessment of the isometric back strength is taken. A written explanation of one of the questionnaires, in which patients have to choose the three most disabled daily activities due to back pain, is given them to take home, which allows them to make a well-considered choice at the next visit. Written informed consent is obtained from all patients who are willing and eligible to participate. At the second visit, participants undergo a baseline measurement consisting of a compound questionnaire and an isometric back strength test. At the third visit, patients are randomized into either the back strengthening group or usual care group. Directly after randomization, the first treatment session starts. Patients are allowed to withdraw from the study at any time, although the importance of full compliance of every participant is emphasized.

Randomization is done by means of a computer-generated table of random numbers per study center, using a block size of ten. Prestratification is applied for the duration of the back complaints, with a cut-off point of 12 weeks, suspecting duration of complaints to influence the individual response to the exercise program. One intervention group receives a back-strengthening program; the other group receives a usual care program.

The randomization is concealed, which means that the treating physiotherapist obtains the allocated treatment by means of a computer software program, by entering the name and military registration number of the patient.

The study protocol was approved by the Medical Ethics Committee of the Netherlands Central Military Hospital.

### Study interventions

#### Back strengthening program

Subjects allocated to the back-strengthening program (BS) undergo a 10-week, progressive resistance-training program of the isolated lumbar extensor muscle groups. The program includes 14 training sessions (2 days per week) and 3 isometric back strength tests (in week 1, 5, and 10). The initial training load is set at approximately 35% of the maximal isometric back extension strength of the participant, measured at baseline. The goal of every training session is to perform 15 to 20 repetitions (reps) on the lower back machine, equivalent to approximately 50% and 70% of the one-repetition maximum (1 RM) respectively. If the subject is able to perform a higher number of reps, 2 1/2 kg weight is added in the next training session. Vice versa, if the participant is unable to perform the minimal number of reps, the subsequent training load is lowered with 2 1/2 kg. This training protocol is partly based on existing protocols [5,18], and partly on our own experiences. A comprehensive training protocol can be obtained from the authors.

Training sessions are carried out on a Total Trunk Rehab (Technogym Inc, Italy). This lower back machine is equipped with a knee-lock system and a thigh-restraining belt to immobilize both hips and thighs, allowing the participant only to move the isolated lower back.

All training sessions are conducted as much as possible by the same physiotherapist. The physiotherapist pays special attention to the execution of the training in terms of pace and movement. The flexion and extension of the lower back has to be executed in the full range of motion of the participant, and movements have to be slow and controlled (moving in two seconds from maximal flexion to maximal extension when lifting the weight stack, and returning from maximal extension to maximal flexion in four seconds when lowering the weight stack). During this movement, emphasis is put on the hollowing and flattening of the lumbar lordosis. Every training session is preceded by a 5-minute all-body warming-up on an arm/leg ergometer (Schwinn Airdyne Pro, Balans Inc., Nieuwegein, The Netherlands). The weight load used and the number of reps completed during each training session are recorded.

#### Usual care program

Subjects allocated to the usual care program (UC) receive regular physical therapy for their lower back for at most 10 weeks, or earlier when the patient indicates to be free of complaints. Based on the physiotherapist's judgment, this could include 'hands-on' treatment (e.g. passive mobilizing and pain-cushioning techniques, manual therapy) and/or 'hands-off' treatment (e.g. exercise therapy, individual education and instruction on the back function). In the RNLA, active therapy forms are favored. To increase the contrast between both intervention programs, physiotherapists are not allowed to use the lower back machine in their usual care. Patients are not allowed to undergo co-treatment beside the interventions programs during the treatment period, nor exercise on equipment that mimics the specific components of the lower back machine.

Therapeutic activities in every therapy session as well as the number of sessions are written down on a form.

### Outcome measurements

In this study we have chosen primary outcome measures that are most relevant to the patient and clinician: self-assessed degree of complaints and degree of handicap in daily activities due to LBP. In addition, our secondary measurements focus on several other LBP-related areas, like kinesiophobia or mental health perception.

We have included some potential prognostic factors into our measurements, i.e. characteristics of the patient that possibly influence the effect of the intervention: job characteristics, overall health, physical activity, patient satisfaction with the allocated treatment, and attitudes and beliefs of the physiotherapist. This trial is mainly focused on the efficacy of our minimal intervention strategy, and not on unraveling the physiological or psychological working mechanisms of isolated lumbar extension training on LBP. Nevertheless, by including potential prognostic variables, starting points for further research into the 'black box' of this type of intervention could be identified.

#### Baseline characteristics

The following demographic variables are registered during the intake: age, time since first episode of LBP, pain radiation, treatment history, and work absenteeism due to LBP in the last year.

Moreover, the status of the patient before treatment, in terms of job aspects, overall health and the degree of physical activity, is assessed using a compound questionnaire. Several job characteristics, i.e. content, relation with superior and colleagues, conflicts, and physical aspects of the job, are measured using subscales of a validated Dutch version of the Job Content Questionnaire [16]. Physical aspects of former jobs are assessed using one item of the Dutch Musculoskeletal Questionnaire [17].

Overall health is assessed with one item from the MOS 36-item Short Form Health Survey [18]: "What do you think, in general, of your health?" (1 = bad, 2 = moderate, 3 = good, 4 = very good, 5 = excellent).

The degree of physical activity is measured with the Short Questionnaire to Assess Health Enhancing Physical Activity [19], a validated and fairly reliable 5-item questionnaire.

To assess the attitudes and beliefs of the participating physiotherapists about the relationship between low back pain and function before the start of the study, we use the Pain Attitudes and Beliefs Scale for Physiotherapists [20]. These attitudes and beliefs are believed to influence the physiotherapist's commitment to a certain treatment approach.

#### Primary outcome measures

Global perceived effect [21] is measured by self-assessment on a 7-point scale (1 = completely recovered, 2 = much improved, 3 = slightly improved, 4 = no change, 5 = slightly worsened, 6 = much worsened, 7 = vastly worsened). We defined scores of 1 and 2 as a clinically important change.

Patient-specific functional status [22] is measured by a questionnaire following a patient-specific approach. At baseline, individual patients select three main complaints, i.e. frequent activities which they perceive as important in their daily life, but which are hampered by their back pain. Patients rate the severity of these three complaints on a 100 mm visual analogue scale at each test moment. The responsiveness of the questionnaire is fairly good, with an area under the ROC-curve of 0.82, and with mediate correlations with the Roland Disability Questionnaire (r = 0.69–0.75) and the Visual Analogue Scale (r = 0.70–0.80) [21].

Low-back specific functional status is measured by the validated Dutch version of the Roland Disability Questionnaire [23,24], a widely used 24-item scale that reflects the functional disability due to LBP. Test-retest reliability of this scale is considered good for three weeks (r = 0.83) and six months (r = 0.72) respectively [25].

#### Secondary outcome measures

Fear of movement or re-injury is measured by the Tampa Scale for Kinesiophobia [26], a 17-item scale to obtain a score for the extent to which a chronic back patient fears (new) physical damage due to physical activity. The Dutch version of this questionnaire has been found sufficient reliable and valid [27,28].

Mental health is measured by the Dutch translation of the 12-item General Health Questionnaire [29,30], assessing problems concerning psychological distress, like depression, sleep deprivation, stress coping, and self confidence. Test-retest reliability of the scale is high in a general population, with reported Cronbach's alpha coefficients between 0.86 and 0.90 [30].

Social health is measured by a subscale of the Impact on Participation and Autonomy Questionnaire [31], focusing on the influence of the disability on social relationships; Cronbach's alpha for this factor is 0.87.

Overall work status is measured by a 4-level item (1 = "currently working full duties and/or able to do all of my regular home duties", 2 = "able to do all work and/or home duties but it causes extra pain", 3 = "on restricted duties at work and/or need help with some of my home duties", 4 = "off work and/or need help with most of my home duties").

Individual back extension strength progression was evaluated using repeated isometric measurements on the lower back training and testing machine. A detailed description of these measurements can be find elsewhere [15].

Patient satisfaction is measured at the end of the treatment program by two 3-level items and one 5-level item, in which the degree of satisfaction with the allocated treatment is assessed: (a) "Were you satisfied with the allocated treatment at the start of the program?" (b) "Has your opinion about the treatment changed during the program?" (c) "How satisfied are you now about the treatment that was given to you?"

### Short- and long-term follow-up

Beside a baseline measurement, follow-up data are gathered at two short-term intervals and two long-term intervals. Short-term follow-up measurements are at 5 and 10 weeks after randomization. Long-term follow-up measurements are at 6 months and one year after the end of the intervention, respectively. At every test moment, participants fill in a compound questionnaire on a stand-alone PC, and they undergo an isometric back strength measurement on the lower back machine. The content of the questionnaire varies per test moment: parts of the questionnaire referring to potential prognostic variables (job characteristics, overall health, and physical activity) are only displayed at baseline, and at 6 and 12 months of follow-up. Figure 1 presents a flow chart of the different phases of the study.

### Blinding

Double blinding or placebo control is virtually impossible in trials that involve treatment modalities like the ones used in this study, since both patient and provider are inevitably aware of the content of the treatment.

Because our physiotherapists are aware of which treatment they provide, there is always the possibility that they may inadvertently convey different expectations to the patients in each treatment program. This could enhance non-specific effects in either of the two groups. Therefore, as mentioned, beliefs and attitude of the physiotherapists towards back treatment in general are evaluated at baseline, as well as at the end of the study period.

Another limitation of trials comparing a relatively new treatment to the usual care is the risk of a potential nocebo effect; i.e. patients might feel disappointed after being allocated to the standard therapy. To minimize this effect, both programs are introduced to the patient as potentially equally effective treatments in restoring back function, with the relative efficacy of both programs as the main focus of the study. Moreover, at baseline patients are informed about the opportunity they have to continue with a treatment modality of their choice (e.g. our training device) after finishing the treatment period. Patient satisfaction with the allocated treatment is measured directly after the treatment period.

Other efforts to achieve a certain level of blinding within patients, physiotherapists, and data researchers, are:

• low back strength training and measurement are done as much as possible by two different physiotherapists;

• an independent data manager collects data from all study locations, and recodes patients and locations to unique codes before handing the database to the researchers.

### Statistics

#### Sample size estimates

We attempt to enroll 200 patients at the four military health centers, i.e. 100 patients per treatment group. According to power calculations (α = 0.05 and 1-β = 80%), this sample size is sufficient to detect a 20% difference in our primary outcome measure Global Perceived Effect, between the BS and UC program. In our beliefs, a 20% difference reflects a clinical relevant change in health status.

#### Data analysis

Statistical analysis will be performed according to the intention-to-treat principle; i.e. patients will be analyzed in the treatment group to which they were randomly allocated. In addition, a per-protocol analysis will be performed, in which only patients with no major protocol deviations will be analyzed. Comparing these analyses with the results of the intention-to-treat analysis will indicate, to what extend protocol deviations and lack of compliance might have biased the results.

In our analyses, we will compare the size of the effect, if any, of isolated back strengthening and of usual care for the low back on our primary and secondary outcome variables. Further analysis will determine whether several potential prognostic variables will influence the magnitude of the treatment responses, and if subsets of patients can be distinguished that can be indicated as good-/bad-responders to our specific back training. Demographic and clinical characteristics, as well as baseline outcome measures will be summarized by descriptive statistics. Longitudinal multilevel analyses will be used to examine differences in all continuous outcome measures at 5 and 10 weeks after randomization, and 6 and 12 months of follow-up.

### Supervision of the centers

In order to obtain full commitment from the participating military health centers, as well as to make sure that every center uses the study protocols in the same way, we organize several feedback and feedforward sessions with all participating physiotherapists. In these sessions, we explain different aspects of the trial design, give instructions on the test and training protocols, and answer remaining questions.

After these sessions, we install the required equipment (test/training machine, soft- and hardware) at the four locations. Each center has a practice period of about 8 weeks to become familiar with the instruments, logistics and protocols, by means of ad hoc training and test sessions with non-participating (regular) back patients. Our researchers join these sessions and, if necessary, give correcting feedback during and after a test or training.

After this practice period, each location officially starts with the study. Once every two weeks, our researchers visit the centers to monitor progress in recruitment and data handling, and to observe the execution of test and training sessions.

## Discussion

This article describes the rationale and design of a multicenter randomized controlled trial in which the efficacy of a specific type of lumbar extension training and usual care are compared in patients with nonspecific LBP longer than 4 weeks. A substantial number of trials have been conducted that included lumbar extension training in low back pain patients [4,7,11,12,32,33]. So far, only the study by Risch et al [11] has emphasized a minimal intervention approach comparable to ours, which was, however, conducted in a non-randomized study design.

Our population at risk can be seen as a selected population of mainly male employees, who work in a dynamic organization with a strong culture of physical fitness. We realize that this selection might limit the external validity of the outcomes of this study. However, results of the trial may be extrapolated to other working populations with a more-than-average degree of physical straining job activities, e.g. policemen and firemen on duty or construction workers.

Besides, despite the "fit-and-healthy" image that soldiers have in our society, a large health survey among a cross-section of male military personnel of 30–40 years showed no favorable scores on several cardiovascular and fitness parameters in comparison to other populations of Dutch men [34,35]. In this perspective, the RNLA – with over 30,000 military and civilian employees a major professional organization in the Netherlands – seems to be a good reflection of Dutch society in general with regard to general health parameters.

Moreover, for reasons of homogeneity it might be even an advantage to only have a study population of working men with (probably) moderate low back trouble in this study.

We hope with this trial to give greater insight to caregivers within and outside the RNLA on treatment options for workers in the sub-acute or chronic phase of their LBP. If, for instance, our minimal intervention approach is equally or more effective than the usual care, medical decision makers may consider implementing this treatment modality in the daily practice of the physiotherapist, by weighing the costs (e.g. price and depreciation costs of materials) and benefits (e.g. reduction of treatment time).

## List of abbreviations

RNLA = Royal Netherlands Army

RCT = randomized controlled trial

LBP = low back pain

BS = Back Strength intervention

UC = Usual Care intervention

rep = repetition on back extension machine

## Competing interests

The authors declare that they have no competing interests.

## Pre-publication history

The pre-publication history for this paper can be accessed here:



## Figures and Tables

**Table 1:** In- and exclusion criteria of the study

**Inclusion criteria:**

•military employees of the RNLA between the age of 18 and 54 years •at least 4 weeks of continuous or recurrent (at least 3 times a week) episodes of LBP pain localized •between posterior iliac crests and angulus inferior scapulae •availability to visit the local military health center 2 times a week during 10 consecutive weeks, with •no more than two sessions of absence due to job-related activities (e.g. military exercise, course, leave) •willingness to abandon other treatment interventions for the lower back during the intervention period •signed informed consent
**Exclusion criteria**
•spinal surgery in the last 2 years •specific treatment for LBP in the last 4 weeks (e.g. physiotherapy, manual therapy) •severe LBP which hinders in performing maximal isometric strength efforts •specific LBP, defined as herniated disc, ankylosing spondylitis, spondylolisthesis or other relevant •neurological diseases
